# A Birth-cohort testing intervention identified hepatitis c virus infection among patients with few identified risks: a cross-sectional study

**DOI:** 10.1186/s12879-015-1283-3

**Published:** 2015-12-01

**Authors:** William N. Southern, Brianna Norton, Meredith Steinman, Joseph DeLuca, Mari-Lynn Drainoni, Bryce D. Smith, Alain H. Litwin

**Affiliations:** Division of Hospital Medicine, 111 East 210th Street, Bronx, 10467 NY USA; Montefiore Medical Center, 111 East 210th Street, Bronx, NY 10467 USA; Division of General Internal Medicine, 111 East 210th Street, Bronx, 10467 NY USA; Section of Infectious Diseases, Department of Medicine, Boston University School of Medicine, 72 East Concord Street, Boston, MA 02118 USA; Division of Viral Hepatitis, Centers for Disease Control and Prevention, National Center for HIV/Viral Hepatitis/STD/TB Prevention, 1600 Clifton Road, Atlanta, GA 30329 USA; Department of Medicine, Montefiore Medical Center, 111 East 210th Street, Bronx, NY 10467 USA

**Keywords:** Hepatitis C virus, Screening, Testing strategies, Risk assessment

## Abstract

**Background:**

International guidelines and U.S. guidelines prior to 2012 only recommended testing for hepatitis C virus (HCV) infection among patients at risk, but adherence to guidelines is poor, and the majority of those infected remain undiagnosed. A strategy to perform one-time testing of all patients born during 1945–1965, *birth cohort testing*, may diagnose HCV infection among patients whose risk remains unknown. We sought to determine if a birth-cohort testing intervention for HCV antibody positivity helped identify patients with fewer documented risk factors or medical indications than a pre-intervention, risk-based testing strategy.

**Methods:**

We used a cross-sectional design with retrospective electronic medical record review to examine patients identified with HCV antibody positivity (Ab+) during a pre-intervention (risk-based) phase, the standard of care at the time, vs. a birth-cohort testing intervention phase. We compared demographic and clinical characteristics and HCV risk-associated factors among patients whose HCV Ab + was identified during the pre-intervention (risk-based testing) vs. post birth-cohort intervention phases. Study subjects were patients identified as HCV-Ab + in the baseline (risk-based) and birth-cohort testing phases of the Hepatitis C Assessment and Testing (HepCAT) Project.

**Results:**

Compared to the risk-based phase, patients newly diagnosed with HCV Ab + after the birth-cohort intervention were significantly less likely to have a history of any substance abuse (30.5 % vs. 49.5 %, *p* = 0.02), elevated alanine transaminase levels of > 40 U/L (22.0 % vs. 46.7 %, *p* = 0.002), or the composite *any risk-associated factor* (55.9 % vs. 79.0 %, *p* = 0.002).

**Conclusions:**

Birth-cohort testing is an useful strategy for identifying previously undiagnosed HCV Ab + because it does not require providers ask risk-based questions, or patients to disclose risk behaviors, and appears to identify HCV **Ab +** in patients who would not have been identified using a risk-based testing strategy.

## Background

An estimated 3.2 million persons are infected with the hepatitis C virus (HCV) in the U.S [1, 2]. In the absence of testing, care, and treatment, HCV infection is predicted to cause 1.5 million cases of cirrhosis and contribute to 900,000 deaths over the lifetime of infected persons [3]. Curative treatment for HCV infection is available, and early testing is associated with early entry into care [4–8]. Previous U.S. and many international guidelines recommend testing patients at risk for HCV infection including those with a history of injection drug use, recipients of transfusions or organ transplants, and those with elevated alanine aminotransferase (ALT) levels [7–12]. However, providers are often non-adherent to risk-based testing guidelines [13], and the majority of those infected remained undiagnosed [13–15].

Data have demonstrated that approximately 77 % of all HCV-infected persons in the U.S. were born between 1945 and 1965 [16] and adults in this cohort have a HCV infection prevalence of 3.2 %, approximately five fold higher than other adults outside of this age cohort [17]. Therefore, in the U.S. one-time testing of all patients born during 1945–1965, birth-cohort testing, is now recommended by the Centers for Disease Control and Prevention and the US Preventive Services Task Force in addition to risk-based testing [18–20]. In addition, it is possible that a birth-cohort-based testing strategy is more effective for identification of HCV-infected patients who have no known risk factors or medical indications for HCV testing. To investigate this, we examined the characteristics and risk factors of HCV- Ab + persons who were identified during a pre-intervention phase when risk-based testing was the standard of care vs. after a birth-cohort testing intervention. We hypothesized that patients identified as HCV- Ab + using a birth-cohort testing strategy would be less likely to have documented risk factors or medical indications as compared to patients identified as HCV- Ab + by traditional risk-based testing strategy.

## Methods

### Study setting

The study was conducted at three community-based primary care clinics affiliated with Montefiore Medical Center, a regional healthcare system including four hospitals and twenty three outpatient sites, located in the Bronx, New York. The estimated prevalence of HCV Ab + in the population served by the study sites is 7.7 % [13].

### Study design and population

Study subjects were drawn from the baseline (risk-based testing) and birth-cohort testing phases of the Hepatitis C Assessment and Testing Project (HepCAT), a cross-sectional intervention study investigating a birth cohort testing strategy for HCV Ab+, described previously [13, 21, 22]. In short, patients were eligible to be included in the risk-based testing group if they had a clinic visit at one of the study sites during the baseline phase of HepCAT (1 January 2008 to 29 February 2008). Patients were eligible to be included in the birth-cohort testing group if they were tested at a clinic visit during the birth-cohort phase of HepCAT (9 March 2009–30 June 2009). The birth-cohort intervention phase was conducted for four months to maximize the number of patients who were tested for HCV. Patients were considered newly-identified HCV- Ab + and included in this study if they had no previous record of HCV Ab+, and tested positive for HCV antibody, at any hospital or site in the Montefiore system, within 2 years before or 90 days after the qualifying clinic visit for the baseline phase or within 90 days after the clinic visit date in the birth-cohort (intervention) phase. Retrospective electronic medical record (EMR) review was used to examine clinical characteristics and HCV risk factors of patients identified with HCV Ab + during the pre-intervention phase (when risk-based testing was the standard of care), vs. after a birth-cohort testing intervention.

### Data extraction

For each research subject we extracted data from Montefiore’s Clinical Information System including demographic information associated with the qualifying clinic visit, and clinical information dating back to March 1997, from any contacts the subjects had with a Montefiore hospital or outpatient site. This included inpatient and outpatient ICD-9 diagnosis codes, prescription and inpatient medication records, and laboratory testing results.

### Birth-cohort vs. Risk-based testing

**Fig. 1:**
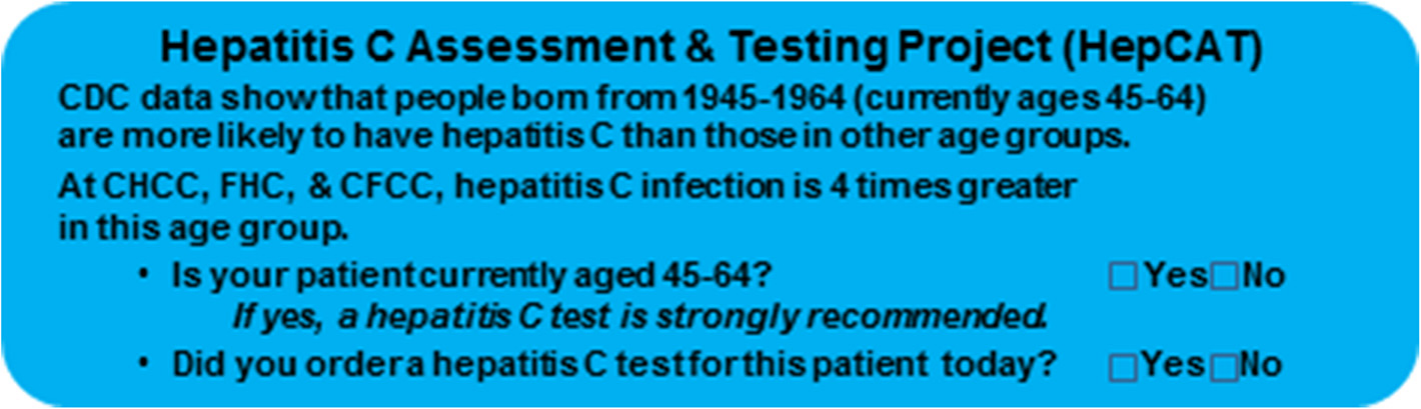
The birth-cohort reminder sticker placed in the chart during the birth-cohort testing phase of the study

During the baseline (pre-intervention) phase of the parent study most patients tested for HCV antibody had a documented risk factor or medical indication suggesting that providers were following risk-based testing recommendations for HCV testing [13]. During the birth-cohort phase, a reminder sticker was placed on each progress note for every visit (Fig. 1). The reminder recommended that providers order an HCV antibody test for all patients born during 1945–1964, regardless of any other identified risk.

### Markers of risk/definitions

Because we were unable to directly measure primary risks for HCV exposure (e.g. injection drug use) we measured factors/markers that may be indicative of being at increased risk for HCV exposure and other demographic characteristics (such as age and race/ethnicity) previously shown to be associated with HCV antibody positivity. A factor/marker for testing was considered present if it appeared anytime in the medical record before the index clinic visit. Independent variables included age, sex, race/ethnicity, any substance abuse (coded as present if an ICD-9 code for substance abuse/dependence or a positive urine toxicology for amphetamines, barbiturates, cocaine, or methadone was recorded), HIV infection (ICD-9 code or positive antibody test confirmed by western blot), sexually transmitted infection [23, 24] (ICD-9 code indicating gonorrhea or chlamydia or positive gonorrhea or chlamydia PCR probe), alcohol abuse [25, 26] (ICD-9 code for alcohol dependence or alcohol-related liver disease, or a serum alcohol level ≥ 80 mg/dl), cirrhosis (ICD-9 code for cirrhosis), end stage renal disease (ICD-9 code for end-stage renal disease or procedure code for hemodialysis), psychiatric disease [27, 28] (ICD-9 code for affective disorder, anxiety disorder, schizophrenia, or psychosis), and ALT elevation (using the highest ALT value reported, considered elevated if > 40 U/L) [29, 30]. In addition, we examined a composite variable, *any risk-associated factor,* which included history of *any* of the aforementioned variables, substance abuse, HIV infection, sexually transmitted infection, alcohol abuse, cirrhosis, end stage renal disease, history of psychiatric illness, or ALT > 40 U/L. Each diagnosis was represented by a group of ICD-9 codes using the classification system of the Healthcare Cost and Utilization Project of the Agency for Healthcare Research and Quality system [31] (Appendix: Table 2).

### Statistical analysis

To determine if patients identified as HCV Ab + during the pre-intervention vs. post intervention phases had different risk profiles, we compared demographic and HCV risk characteristics between the two groups using *t*-tests, Chi-squared and Fisher’s exact tests, as appropriate. A *p*-value < 0.05 was considered significant.

STATA/IC software, version 10.0, (StataCorp, College Station, TX) was used for all data management and statistical analysis. The Institutional Review Boards of Boston University Medical Center and Montefiore Medical Center approved this study.

## Results

The study sample included 164 patients who were newly identified HCV Ab+. The study population mean age was 49.9 +/− 12.0 years, 57.9 % male, 60.4 % Latino, 26.2 % non-Hispanic Black and 4.9 % non-Hispanic White. Of these, 105 patients (64.0 %) were identified HCV Ab + during the risk-based phase and 59 patients (36.0 %) during the birth-cohort phase. Patients identified during the birth-cohort phase were less likely to be male (49.1 % vs. 62.9 %, *p* = 0.09), but the difference was not significant. The patient groups were similar with respect to age and racial/ethnic characteristics.

Compared to the risk-based phase, patients identified during the birth-cohort phase were significantly less likely to have a documented history of substance abuse (30.5 % vs. 49.5 %, *p* = 0.02), an ALT measurement > 40 U/L (22.0 % vs. 46.7 %, *p* = 0.002), or the composite *any risk-associated factor* (55.9 % vs. 79.0 %, *p* = 0.002). In addition, other co-morbidities were less common in the birth-cohort group, including HIV infection (10.2 % vs. 19.0 %, *p* = 0.13), cirrhosis (0.0 % vs. 4.7 %, *p* = 0.16), and history of psychiatric illness (11.9 % vs. 21.9 %, *p* = 0.11), but the differences were not significant (Table 1).

**Table 1:** Characteristics of Patients Identified HCV Positive using Risk-based vs. Birth-cohort Strategies

	Risk-based phase	Birth-cohort phase	
	*n* = 105 (%)	*n* = 59 (%)	*p* value
Age	50.5 ± 10.8	48.9 ± 12.0	0.45
Male	66 (62.9)	29 (49.2)	0.09
Race/Ethnicity			0.99
White	5 (4.8)	3 (5.1)	0.99
Black	27 (25.7)	16 (27.1)	0.84
Hispanic	64 (61.0)	35 (59.3)	0.84
Other/Unknown	9 (8.6)	5 (8.5)	0.98
Insurance			0.66
Medicare	5 (4.8)	5 (8.5)	0.34
Medicaid	83 (79.0)	42 (71.2)	0.26
Commercial	8 (7.6)	5 (8.5)	0.85
Self	9 (8.6)	7 (11.9)	0.49
History Substance Abuse	52 (49.5)	18 (30.5)	0.02
HIV Infection	20 (19.0)	6 (10.2)	0.13
History STI^a^	2 (1.9)	0 (0.0)	0.54
Alcohol Abuse	4 (3.8)	1 (1.7)	0.65
Cirrhosis	5 (4.7)	0 (0.0)	0.16
End Stage Renal Disease	0 (0.0)	2 (3.4)	0.13
History Psychiatric Illness	23 (21.9)	7 (11.9)	0.11
ALT > 40 U/L	49 (46.7)	13 (22.0)	0.002
Any Factor^b^	83 (79.0)	33 (55.9)	0.002

^a^STI: Sexually Transmitted Infection

^b^Any Factor: History Substance Abuse, HIV Infection, Sexually Transmitted Infection, Alcohol Abuse, Cirrhosis, End Stage Renal Disease, History of Psychiatric illness, or ALT > 40 U/L

## Discussion

We found that patients who were newly identified as HCV Ab + after a birth-cohort testing intervention were significantly less likely to have a documented indication for HCV testing than patients identified during the risk-based testing phase. Specifically, birth-cohort phase patients were significantly less likely to have a documented history of substance abuse or elevated serum ALT levels. In addition, it was less common for birth-cohort phase patients to have one of several diagnoses that might trigger and HCV test including of HIV infection, cirrhosis, or a history of psychiatric illness. Our study highlights an important limitation of a risk-based testing strategy and suggests that providers using a birth-cohort testing strategy may find HCV infection among a number of patients without an identified risk or clinical indication for testing whose infection would not have been found otherwise.

Though risk-based testing is important given the high prevalence of disease among patients with known risk factors, this testing strategy has had limited effectiveness. The majority of the HCV-infected individuals in the United States are still unaware of their infection 25 years after the discovery of the hepatitis C virus [14, 15]. Risk-based testing is limited because it is dependent on the physician’s willingness and time to inquire about risk, as well as a patient’s ability to recall risk exposure, such as a blood transfusion prior to 1992, or comfort with disclosing risk, such as injection drug use (IDU) [32]. As a result, risk-based testing may not identify HCV-infected patients despite exposure to risk at some point in their lives. It is likely that most of the patients in the study were exposed to HCV through injection drug use [9]. However, during the birth-cohort intervention phase the vast majority of patients (41 of 59) did not have substance abuse as an identified risk in their medical chart, highlighting the limitations of risk-based testing. A birth-cohort only testing strategy also has limitations: it would fail to identify infection among young people who inject drugs, a critical group in the ongoing spread of HCV infection. For this reason it is likely that a combination of risk-based and birth-cohort testing is optimal in the US.

In addition, elevated ALT levels may be a common reason for testing a patient for HCV, but many patients with HCV infection do not have elevated liver enzymes and their infection may be missed [33, 34]. Our results suggest that the birth-cohort testing strategy is more likely to identify HCV- Ab + individuals without an elevated ALT level. This study demonstrated that individuals living with HCV who do not have known HCV risks may be identified through birth-cohort testing. These individuals will now be able to receive confirmatory testing, and if appropriate, proper counseling, care, and treatment for their infection.

Now more than ever, identification of all HCV-positive individuals is crucial. With the advent of highly effective, tolerable and easily administered HCV medications, we have an opportunity to halt rising morbidity and mortality, and there is long-term potential for eradication of HCV disease [35]. Effective testing remains the first step in this process. As shown in this study, birth-cohort testing may help identify those without known risk factors, improving the detection rates and treatment opportunity for a subset of those currently unaware of their infection. Studies have shown that birth-cohort screening is cost-effective, even in the era of expensive direct-acting antivirals [36], and models have predicted that implementation of this screening strategy could avert 78,000-121,000 deaths, and over 10,000–19,000 liver transplants among those living with HCV who remain undiagnosed [36, 37].

Our study has several limitations. First, the CDC and USPSTF birth-cohort testing guidelines were developed based on HCV prevalences in the U.S and may not be applicable outside the U.S. Whether there are birth-cohort populations outside of the U.S. that would benefit from testing is unknown. Next, we measured HCV antibody positivity, not RNA confirmed HCV infection. In addition, our risk-associated factors were primarily defined by ICD-9 codes which may have resulted in misclassification. Although we believe that this classification is likely non-differential and did not bias our results, we cannot confirm that this is so. Next, we did not measure the adherence to the intervention or the durability of the intervention. In addition, we were not able to directly measure risks for HCV infection (e.g., injection drug use) and so instead measured surrogates for risk that, if noted, might provoke HCV testing. Finally, because our sample size was relatively small we were unable to perform multivariate analyses.

## Conclusions

The majority of HCV-infected persons remain unidentified. Now that curative therapy is available, uptake of Centers for Disease Control and Prevention and US Preventive Services Task Force HCV screening guidelines for birth cohort testing is critical so that HCV-infected individuals with no known risk factors or apparent clinical indications for testing can be identified.

## Appendix

**Table 2:** ICD-9 Code Diagnosis Grouping

Diagnosis grouping	ICD-9 Codes
Substance Abuse	304.0 ,304.00,304.01, 304.02, 304.03, 304.1, 304.10, 304.11, 304.12, 304.13, 304.2, 304.20, 304.21, 304.22, 304.23, 304.3, 304.30, 304.31, 304.32, 304.33, 304.4, 304.40, 304.41, 304.42, 304.43, 304.5, 304.50, 304.51, 304.52, 304.53, 304.6, 304.60, 304.61, 304.62, 304.63, 304.7, 304.70, 304.71, 304.72, 304.73, 304.8, 304.80, 304.81, 304.82, 304.83, 304.9, 304.90, 304.91, 304.92, 304.93, 305.2, 305.20, 305.21, 305.22, 305.23, 305.3, 305.30, 305.31, 305.32, 305.33, 305.4, 305.40, 305.41, 305.42, 305.43, 305.5, 305.50, 305.51, 305.52, 305.53, 305.6, 305.60, 305.61, 305.62, 305.63, 305.7, 305.70, 305.71, 305.72, 305.73, 305.8, 305.80, 305.81, 305.82, 305.83, 305.9, 305.90, 305.91, 305.92, 305.93, 292.0, 292.1, 292.11, 292.12, 292.2, 292.8, 292.81, 292.82, 292.83, 292.84, 292.85, 292.89, 292.9, 648.3
HIV	042, 042.0, 042.1, 042.2, 042.9, 043.0, 043.1, 043.2, 043.3, 043.9, 044.0, 044.9, 079.53, 279.10, 279.19, 795.71, 795.8, V08
Sexually Transmitted Infection	090.0, 090.1, 090.2, 090.3, 090.40, 090.41, 090.42, 090.49, 090.5, 090.6, 090.7, 090.9, 091.0, 091.1, 091.2, 091.3, 091.4, 091.50, 091.51, 091.52, 091.61, 091.62, 091.69, 091.7, 091.81, 091.82, 091.89, 091.9, 092.0, 092.9, 093.0, 093.1, 093.20, 093.21, 093.22, 093.23, 093.24, 093.81, 093.82, 093.89, 093.9, 094.0, 094.1, 094.2, 094.3, 094.81, 094.82, 094.83, 094.84, 094.85, 094.86, 094.87, 094.89, 094.9, 095.0, 095.1, 095.2, 095.3, 095.4, 095.5, 095.6, 095.7, 095.8, 095.9, 096, 097.0, 097.1, 097.9, 098.0, 098.10, 098.11, 098.12, 098.13, 098.14, 098.15, 098.16, 098.17, 098.19, 098.2, 098.30, 098.31, 098.32, 098.33, 098.34, 098.35, 098.36, 098.37, 098.39, 098.40, 098.41, 098.42, 098.43, 098.49, 098.50, 098.51, 098.52, 098.53, 098.59, 098.6, 098.7, 098.81, 098.82, 098.83, 098.84, 098.85, 098.86, 098.89, 099.0, 099.1, 099.2, 099.3, 099.4, 099.40, 099.41, 099.49, 099.50, 099.51, 099.52, 099.53, 099.54, 099.55, 099.56, 099.59, 099.8, 099.9
Alcohol Abuse	291.81, 305.0, 303.0, 303.9, 655.4, 760.71, 291.1, 291.89, 291.2, 425.5, 291.0, 291.1, 291.2, 291.3, 291.82, 291.9, 291.5, 291.9, 291.4, 571.0, 571.3, 571.1, 571.2, 265.2, 980.9, 303.90, 303.91, 303.92, 303.93, 648.4, 760.71, V11.3
Cirrhosis	571.5, 571.2, 571.6, 571
End Stage Renal Disease	585.6
Psychiatric Diagnosis	295.00, 295.01, 295.02, 295.03, 295.04, 295.05, 295.10, 295.11, 295.12, 295.13, 295.14, 295.15, 295.20, 295.21, 295.22, 295.23, 295.24, 295.25, 295.30, 295.31, 295.32, 295.33, 295.34, 295.35, 295.40, 295.41, 295.42, 295.43, 295.44, 295.45, 295.50, 295.51, 295.52, 295.53, 295.54, 295.55, 295.60, 295.61, 295.62, 295.63, 295.64, 295.65, 295.70, 295.71, 295.72, 295.73, 295.74, 295.75, 295.80, 295.81, 295.82, 295.83, 295.84, 295.85, 295.90, 295.91, 295.92, 295.93, 295.94, 295.95, 296.00, 296.01, 296.02, 296.03, 296.04, 296.05, 296.06, 296.10, 296.11, 296.12, 296.13, 296.14, 296.15, 296.16, 296.20, 296.21, 296.22, 296.23, 296.24, 296.25, 296.26, 296.30, 296.31, 296.32, 296.33, 296.34, 296.35, 296.36, 296.40, 296.41, 296.42, 296.43, 296.44, 296.45, 296.46, 296.50, 296.51, 296.52, 296.53, 296.54, 296.55, 296.56, 296.60, 296.61, 296.62, 296.63, 296.64, 296.65, 296.66, 296.7, 296.80, 296.81, 296.82, 296.89, 296.90, 296.99, 297.0, 297.1, 297.2, 297.3, 297.8, 297.9, 298.1, 298.2, 298.3, 298.4, 298.8, 298.9, 298.0, 300.4, 301.11, 301.13, 299.00, 299.01, 299.10, 299.11, 299.80, 299.81, 299.90, 299.91, 300.00, 300.01, 300.02, 300.09, 300.10, 300.11, 300.12, 300.13, 300.14, 300.15, 300.16, 300.19, 300.20, 300.21, 300.22, 300.23, 300.29, 300.3, 300.5, 300.6, 300.7, 300.81, 300.82, 301.0, 301.10, 301.12, 301.20, 301.21, 301.22, 301.3, 301.4, 301.50, 301.51, 301.59, 301.6, 301.7, 301.81, 301.82, 301.83, 301.84, 301.89, 301.9, 307.40, 307.41, 307.42, 307.43, 307.44, 307.45, 307.47, 307.48, 307.49, 307.80, 307.81, 307.89, 307.9, 308.0, 308.1, 308.2, 308.3, 308.4, 308.9, 309.81, 312.30, 312.31, 312.32, 312.33, 312.34, 312.35, 312.39

## Abbreviations

ALT: Alanine aminotransferase
CDC: Centers for Disease Control and Prevention
EMR: Electronic medical record
HCV: Hepatitis C virus
HCV-Ab+: Hepatitis C virus antibody positive
HIV: Human Immunodeficiency Virus
ICD-9: International classification of diseases
IDU: Injection drug use
PCR: Polymerase chain reaction
RNA: Ribonucleic acid
USPS: United States preventive services task force
